# Ensemble Learning of Convolutional Neural Network, Support Vector Machine, and Best Linear Unbiased Predictor for Brain Age Prediction: ARAMIS Contribution to the Predictive Analytics Competition 2019 Challenge

**DOI:** 10.3389/fpsyt.2020.593336

**Published:** 2020-12-15

**Authors:** Baptiste Couvy-Duchesne, Johann Faouzi, Benoît Martin, Elina Thibeau–Sutre, Adam Wild, Manon Ansart, Stanley Durrleman, Didier Dormont, Ninon Burgos, Olivier Colliot

**Affiliations:** ^1^Paris Brain Institute, ICM, Paris, France; ^2^Inserm, U 1127, Paris, France; ^3^CNRS, UMR 7225, Paris, France; ^4^Sorbonne Université, Paris, France; ^5^Inria Paris, Aramis project-team, Paris, France; ^6^Institute for Molecular Bioscience, The University of Queensland, St Lucia, QLD, Australia; ^7^AP-HP, Hôpital de la Pitié-Salpêtrière, Department of Neuroradiology, Paris, France

**Keywords:** brain age, MRI, machine learning, deep learning, statistical learning, ensemble learning

## Abstract

We ranked third in the Predictive Analytics Competition (PAC) 2019 challenge by achieving a mean absolute error (MAE) of 3.33 years in predicting age from T1-weighted MRI brain images. Our approach combined seven algorithms that allow generating predictions when the number of features exceeds the number of observations, in particular, two versions of best linear unbiased predictor (BLUP), support vector machine (SVM), two shallow convolutional neural networks (CNNs), and the famous ResNet and Inception V1. Ensemble learning was derived from estimating weights via linear regression in a hold-out subset of the training sample. We further evaluated and identified factors that could influence prediction accuracy: choice of algorithm, ensemble learning, and features used as input/MRI image processing. Our prediction error was correlated with age, and absolute error was greater for older participants, suggesting to increase the training sample for this subgroup. Our results may be used to guide researchers to build age predictors on healthy individuals, which can be used in research and in the clinics as non-specific predictors of disease status.

## Introduction

Chronological age is an important risk factor for many conditions such as neurological disorders (e.g., Alzheimer's and Parkinson's), chronic (including cardiovascular) disorders, cancer, or stroke, to name a few. However, it is an imperfect predictor of disease risk or of healthy individuals' functional capability (1). A growing field of research has been focusing on identifying biological correlates of age (e.g., from telomere length, methylation site, brain structure, and function) to derive measures of biological age (2–6). Promises of biological age rely on the assumption that it would capture specific physiological or biological aspects of aging, which may allow predicting mortality and could supersede chronological age in predicting diseases or functional state (5, 7). In particular, brain age estimation from MRI images is a rapidly expanding field of research with several hundred publications to date (4).

Predicted age difference (PAD; defined as the difference between predicted age and chronological age) has been associated with mortality and functional measures (6). In addition, brain age (and PAD) trained on healthy participants may be applied to case–control samples where they have been shown to be non-specific predictors of disease status: Alzheimer's disease and conversion (8–10), schizophrenia (11), alcohol dependence (12), cognitive impairment (13), or functional abilities (6, 14). The interested reader may refer to Le et al. (15) and Smith et al. (16) for further discussion on PAD analyses and possible pitfalls. Overall, these results indicate that brain age is associated with disorders, mortality, and function beyond what can be explained by chronological age. In addition, brain age (and PAD) has been shown to be heritable (17, 18), and recent genome-wide association studies (GWASs) have started shedding light on some of the molecular mechanisms responsible for brain aging (19, 20). Lastly, combining brain age and methylation age (21) resulted in an increased prediction of the mortality risk, suggesting that brain age and the epigenetic clock capture different mechanisms of aging (6).

However, the wide range of algorithms that may be used to train brain age predictors, as well as the numerous MRI modalities and processing options [see (4), for a review], raise the question of the robustness of the associations with PAD. In addition, brain age scores are often described by their predictive ability (in predicting chronological age), though comparison of performance across publications is uneasy (4) due to the numerous competing statistics [e.g., mean absolute error (MAE), root mean square error, and Pearson's correlation] and the different datasets used for evaluation.

The Predictive Analytics Competition (PAC) 2019 challenge offers a unique opportunity to benchmark algorithms, techniques (e.g., data augmentation), and image processing options by offering a common framework to all research groups. In short, the test set was not accessible to the participants to avoid overfitting and data leakage, and prediction accuracy was assessed using MAE, while a secondary challenge aimed at also minimizing bias (defined as the association between PAD and chronological age). With the use of the data proposed for the PAC challenge, a previous publication reported a MAE of about 4–5 years (6, 22), in line with the best results reported in the literature at the time (4).

It is important to note that beyond the (methodologically useful) benchmarking of prediction allowed by the PAC challenge, minimizing the prediction error may sound counterintuitive when trying to identify correlates of PAD [see (23), for a real data example]. At the extreme, a perfect age predictor would not provide any additional information than chronological age, even though we do not know whether such a perfect predictor is theoretically possible. On the other hand, minimizing the error bias can guarantee that the age prediction error (PAD, interpreted as accelerated brain aging) is independent of chronological age and, thus, that associations with brain age are not attributable to chronological age differences (15).

Here, we sought to evaluate the performance of different predictive algorithms [convolutional neural networks (CNNs), support vector machine (SVM), and best linear unbiased predictor (BLUP)], as well as their combined predictive accuracy. In addition, we conducted *post hoc* analyses to investigate the effect of (i) MRI processing; (ii) number of models combined; and (iii) site, sex, and age on the brain age prediction accuracy. As a by-product of the BLUP analysis, we also discuss the theoretical maximum prediction that may be achieved from the T1-weighted (T1w) processed images.

## Materials

### Participants From Training Sample

**Figure 1:**
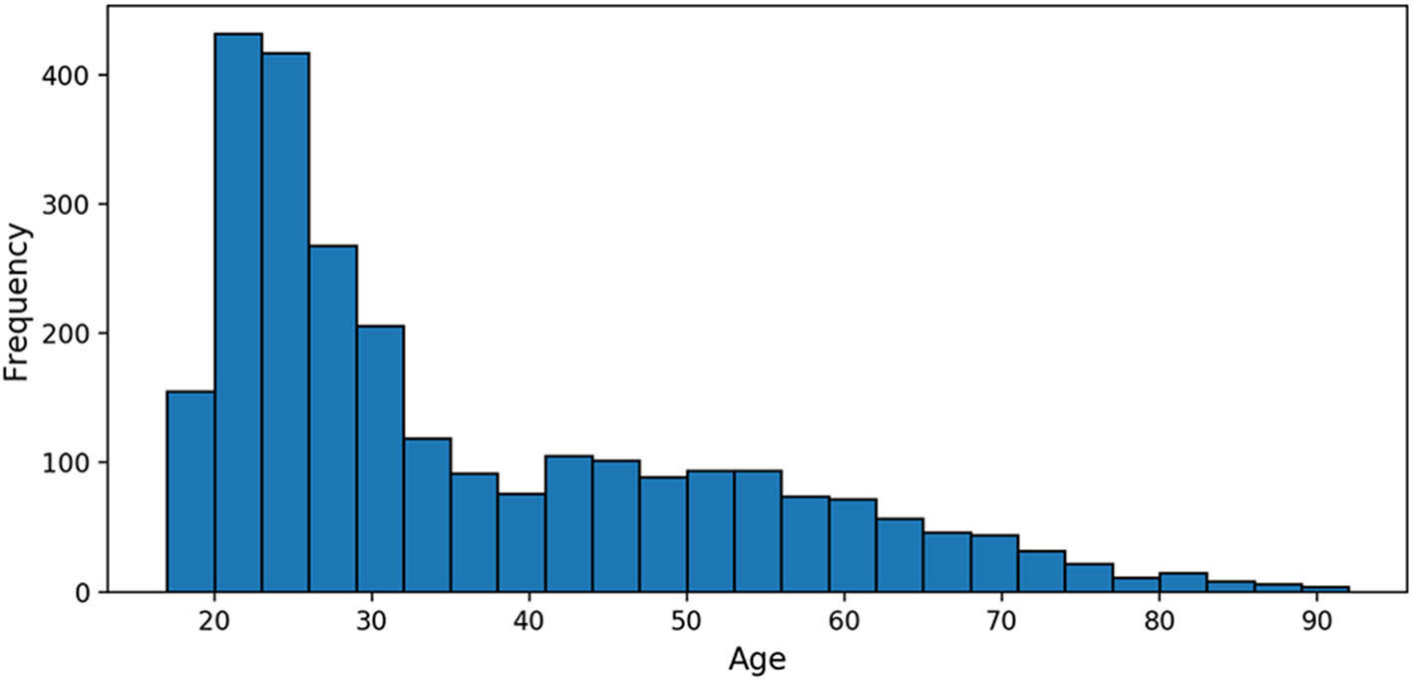
Age distribution of the Predictive Analytics Competition (PAC) 2019 training sample.

The 2,640 PAC participants were 35.8 years old on average (SD = 16.2, range 17–90, Figure 1), imaged across 17 sites; 53% of the participants were females. The smallest site contributed 10 MRIs; the largest 576 (21.8% of the sample). Details about the samples gathered may be found in Cole et al. (17).

## Methods

### Image Features

#### Gray Matter and White Matter Maps

Images were non-linearly registered to the MNI152 space and segmented according to the tissue probability (gray matter, white matter, or cerebrospinal fluid) using SPM12 (University College London, London, UK) and DARTEL (24). A map was produced for each tissue and smoothed using a 4-mm kernel. Gray and white matter maps were distributed by the PAC team; see Cole et al. (17) for details about preprocessing options.

#### Surface-Based Processing of Gray Matter

We manually corrected the orientation of the raw images from site 14, where the axes had been swapped on the raw images. We processed the raw T1w images using FreeSurfer 6.0 to extract vertex-wise measurements of cortical thickness and surface area (*fsaverage* mesh, no smoothing) (25). In addition, we used the ENIGMA-shape protocol (http://enigma.ini.usc.edu/protocols/imaging-protocols/) to further extract a vertex-wise characterization of seven subcortical nuclei thickness and surface (26, 27). Our processing resulted in ~650,000 gray matter measurements per individual; and we previously showed that compared with other surface-based processing, these options maximized the association with age in the UK Biobank (28).

Processing failed for 89 participants (3.4%) from the training sample and 21 (3.2%) from the PAC test set. Most of those individuals belonged to site 8 (76/89 in the training set and 20/21 in the test set) and failed because of white matter segmentation error (topology defects) that we attributed to the lower image quality (after visual inspection of the MRIs).

### Machine Learning Models

#### Overview

We constructed several age predictors, either based on the 3D maps of gray and/or white matter (deep learning models: six-layer CNN, ResNet, and Inception V1) or based on vertex-wise measurements from the surface-based processing (models BLUP and SVM). All algorithms used can derive predictions from a complex image (e.g., high-resolution 3D) or a large number of image-derived measurements (e.g., more features than participants), though only the deep learning approaches leverage the spatial proximity between vertices.

Note that FreeSurfer failed for a handful of participants (see *Surface-Based Processing of Gray Matter*), making BLUP or SVM prediction impossible. To avoid missing values in age prediction, we attributed to those subjects the site- and sex-specific mean age estimated from the training set.

#### Model 1: Best Linear Unbiased Predictor

BLUP scores (29–31) are routinely used in genomics and animal breeding (32, 33) and more recently in neuroscience (28) where the number of features (e.g., single-nucleotide polymorphisms, methylation probes, and vertex measurements) greatly exceeds the number of participants. BLUP scores have the desirable properties of minimizing the mean square error within the class of linear unbiased predictors (30, 31), leading to greater prediction accuracy in genetics (34). In addition, BLUP calculation is computationally efficient, as it does not require hyperparameter estimation. Instead, BLUP uses the estimated variance–covariance matrix between the features (here vertices) to derive the joint marginal associations between the trait and each vertex (30, 31). We used the OSCA software (35) to estimate the BLUP scores. The model used in BLUP calculation also allows estimating the total (linear) association between a trait and features (coined morphometricity), which represents the upper bound of the (linear) prediction accuracy that may be achieved from the data (28, 36).

We scaled the scores using the mean and SD calculated from each site of the discovery sample (BLUP-mean). In addition, and to better account for the non-normal distribution of age in the PAC sample, we also applied a quantile-based scaling by which we forced the predicted age distribution to match that of the training sample (BLUP-quantiles).

#### Model 2: Support Vector Regression

We used SVM (37) with a radial basis function kernel. SVM is a popular machine learning algorithm that was first introduced to address binary classification tasks (38) and then extended to regression tasks. The regression version has yielded successful applications in numerous fields, including time series prediction (39), energy forecasting (40, 41), recognition (42), and medicine (43). We used the implementation provided in the Python package scikit-learn (44).

#### Model 3: Six-Layer Convolutional Neural Networks

The success of CNNs in computer vision has led to numerous applications in medical imaging and more recently in age prediction from neuroimaging data (17, 45–49).

**Figure 2:**
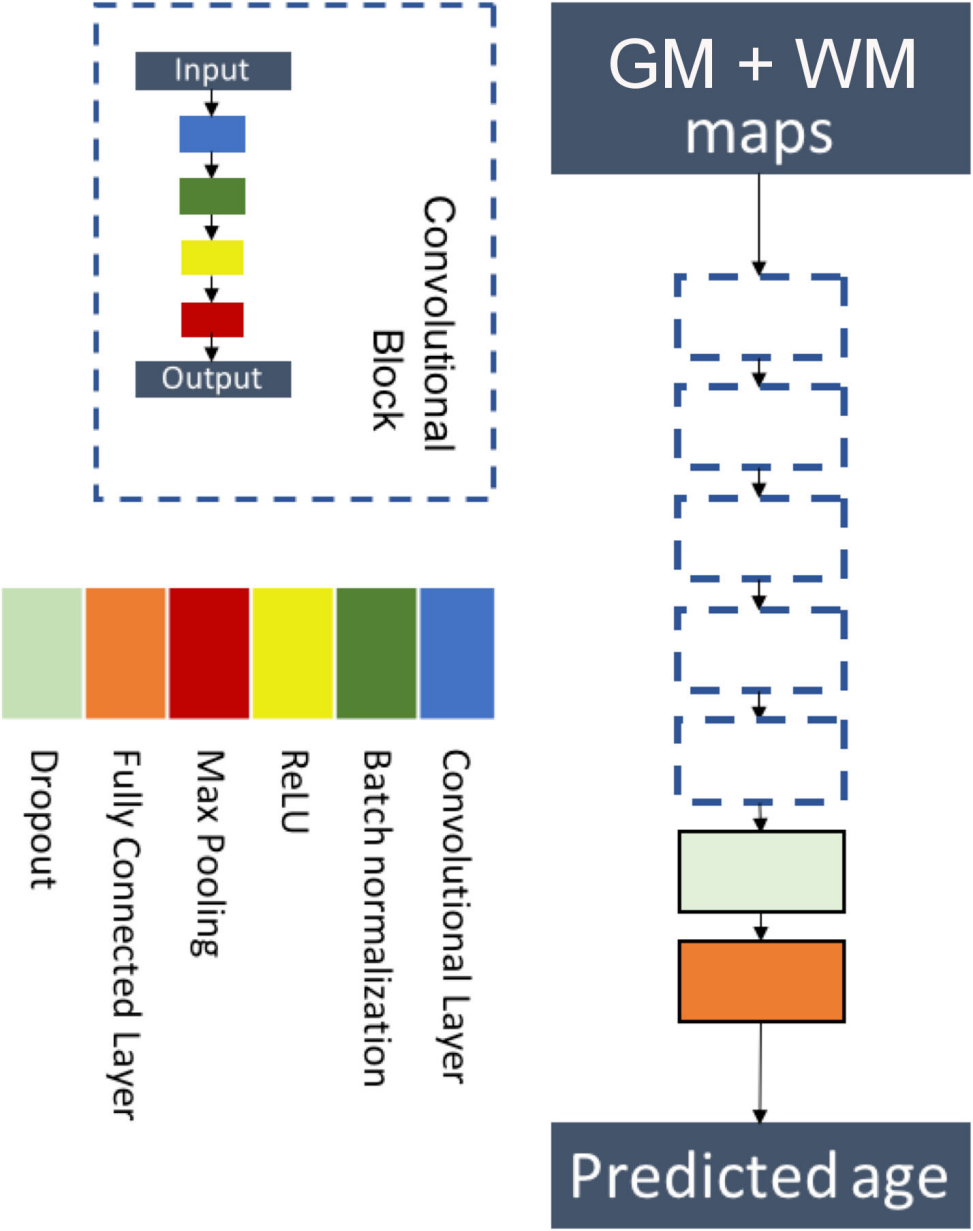
Flowchart showing the components of the proposed six-layer convolutional neural network (CNN) architecture. The network consists of five convolutional blocks followed by a flattening layer and a fully connected layer. Each convolutional block is made of a convolutional layer, a batch normalization layer, a ReLU activation, and a max pooling layer.

We chose a custom architecture with five convolutional blocks followed by a flattening layer and a fully connected layer. Each convolutional block was sequentially made of a convolutional layer, a batch normalization layer, a ReLU activation, and a max pooling layer. This architecture is a simplified version of the architecture of (17) and is displayed in Figure 2. Details on the hyperparameters of the architecture are presented in Supplementary Table 1.

The model was trained using the concatenation of the 3D maps of gray matter and white matter on two channels. We used a MAE loss function, and the model was optimized using Adam (50) with a learning rate of 0.001, a decay of 10^−4^, and setting β_1_ and β_2_ to 0.9 and 0.999, respectively.

#### Model 4: Specialized Six-Layer Convolutional Neural Networks for Younger and Older Subjects

This model is the combination of two CNNs with the architecture described in the previous section. The first CNN was trained on the whole dataset, whereas the second one was only trained on participants older than 40. The age of older participants was given by the mean value of the models, whereas the age of younger ones was given by the first CNN only.

#### Model 5: ResNet

**Figure 3:**
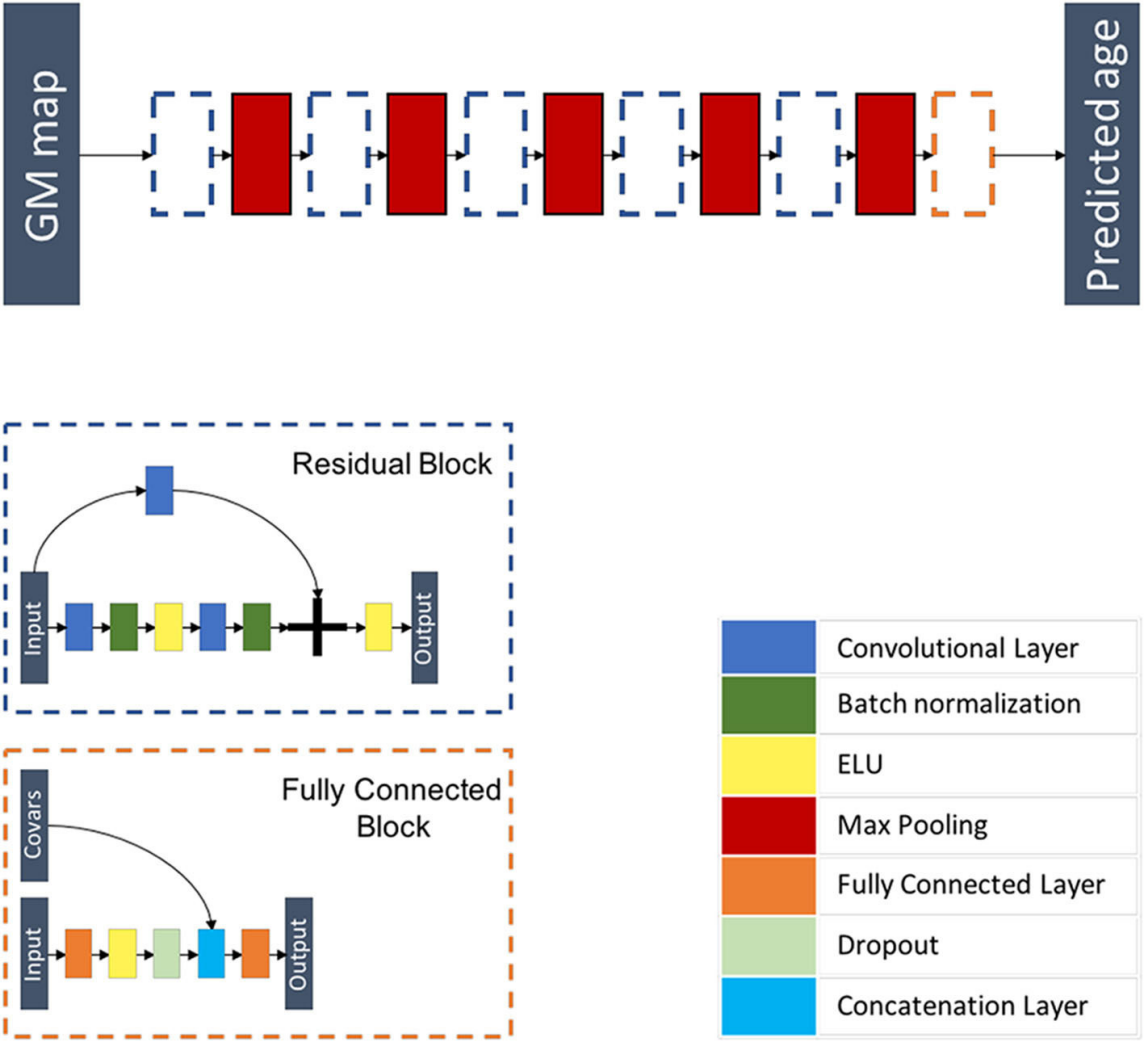
Flowchart showing the components of the proposed ResNet architecture. The network is composed of five residual blocks, each followed by a max pooling layer and a fully connected block. Each residual block is a combination of layers that are repeated twice. Each layer is composed of a 3D convolutional layer, a batch re-normalization layer, and an exponential linear unit (ELU) activation function. A skip connection is added before the last activation function. The fully connected block is composed of a fully connected layer, an ELU activation function, a dropout layer, a layer concatenating additional co-variables, and a fully connected layer.

Inspired from Jonsson et al. (19), this model is a 3D CNN composed of five residual blocks each followed by a max pooling layer of kernel size 3 × 3 × 3 and of stride 2 × 2 × 2, followed by a flattening layer and a fully connected block where additional covariables are concatenated before the last fully connected layer. Each residual block is a combination of layers that are repeated twice. Each layer is composed of a 3D convolutional layer with a kernel size of 3 × 3 × 3 and stride 1 × 1 × 1, a batch re-normalization layer, and an exponential linear unit (ELU) activation function. A skip connection is added before the last activation function. This architecture is summarized in Figure 3 and Supplementary Tables 3, 4.

We trained the model using the 3D maps of gray matter density. We used a MAE loss function and performed optimization using Adam (50) by using the following parameters: a learning rate of 0.0001, a decay of 10^−6^, and setting β_1_ and β_2_ to 0.9 and 0.999, respectively. Our model differed from that of the original paper (19) in that we used a stochastic initialization as opposed to He's initialization strategy (51). In addition, we did not perform data augmentation.

#### Model 6: Inception V1

**Figure 4:**
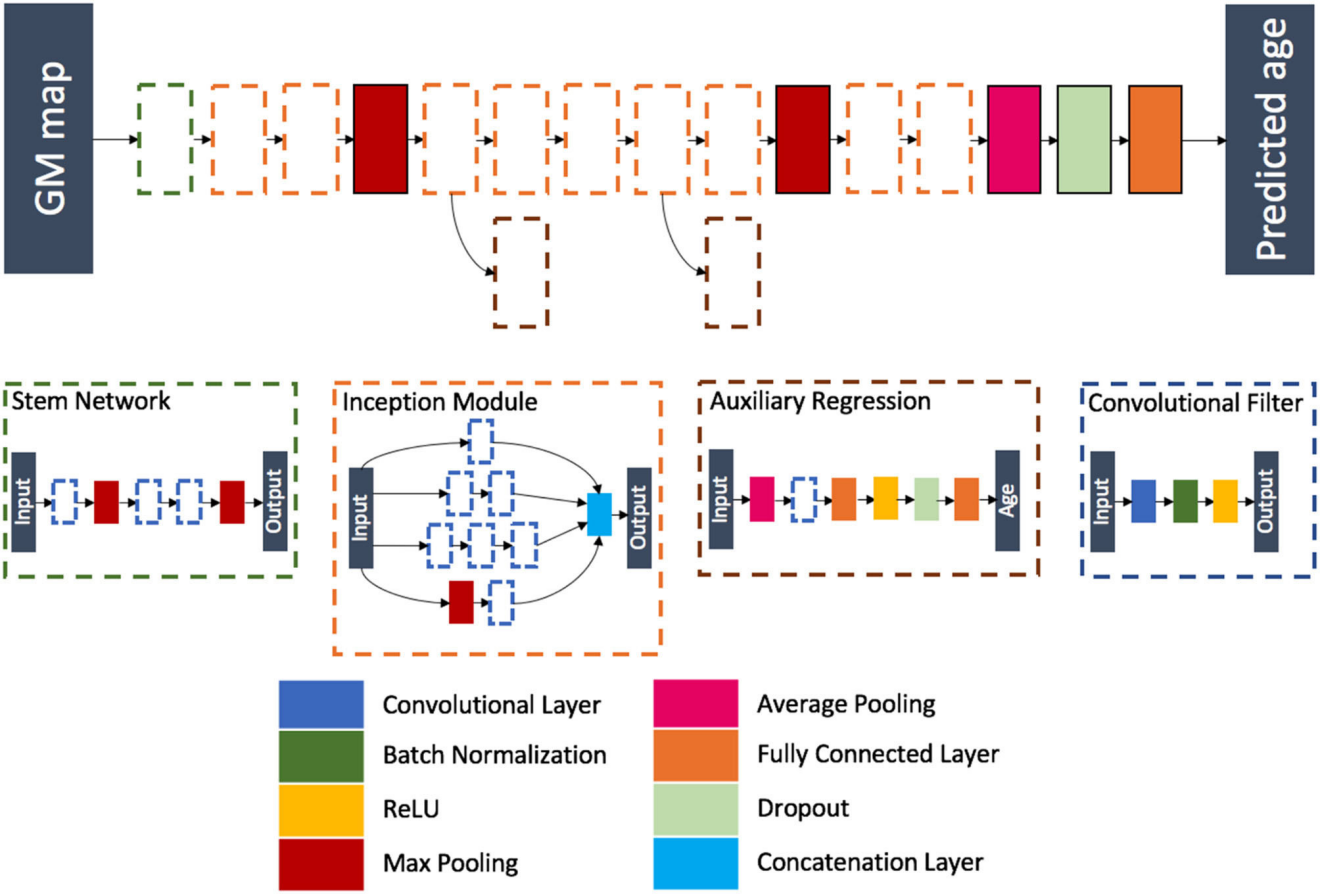
Flowchart showing the components of the proposed Inception architecture. The network is composed of different blocks: a stem network followed by two inception modules, a max pooling layer, five inception modules (two of them being connected to an auxiliary regression), a max pooling layer, two inception modules, an average pooling layer, a dropout layer, and a fully connected layer.

Inspired from the winning architecture for the ILSVRC 2014 competition, this model is a modified version of Google's incarnation of the Inception architecture (52). Our model is able to handle 3D images by using 3D convolution, batch, normalization and pooling layers. The final softmax layer was removed leaving a fully connected layer as the last layer, thus ensuring a regression task instead of a classification task. During training, auxiliary outputs are used to inject additional gradients to mitigate the vanishing gradient problem. Those auxiliary outputs, using fully connected layers for intermediate regression, tend to make the backpropagation computationally infeasible due to the increased number of parameters when going from 2D to 3D. This problem is handled thanks to the regression nature of the problem, as the output dimension is no longer the number of classes but a single real number. We detailed the full architecture in Figure 4 and Supplementary Tables 5–7.

The model was trained using the 3D maps of gray matter density. A MAE loss function was used, and the model was optimized using Adam (learning rate of 10^−4^ and batches of eight images). We kept the model with the highest validation accuracy over 300 epochs.

### Predictive Analytics Competition 2019 Challenge Experiments

#### Training and Validation Procedures

**Figure 5:**
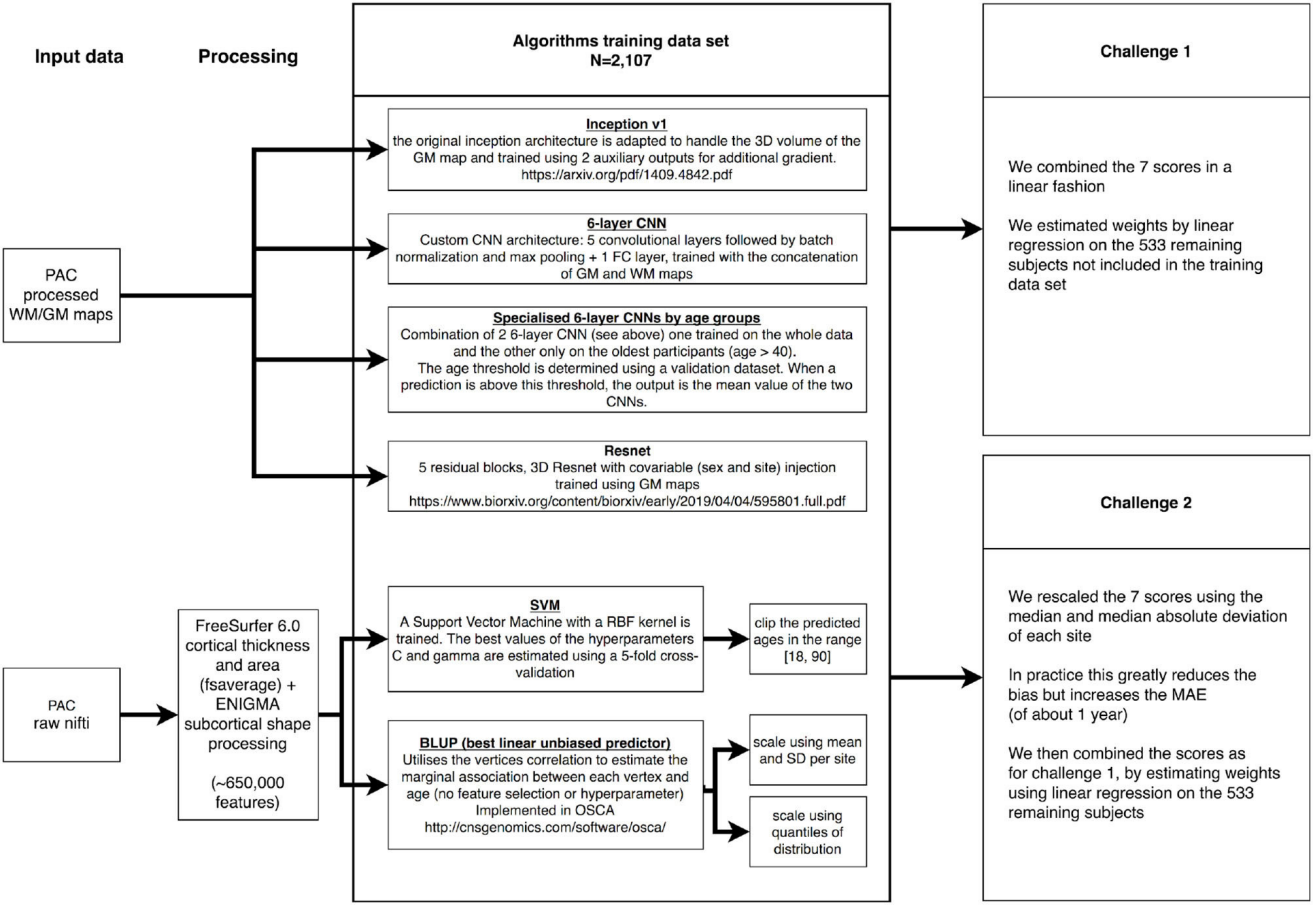
Summary of the Predictive Analytics Competition (PAC) challenge experiments. We present the different data sources, algorithms, and ensemble approaches used in this analysis. GM, gray matter; WM, white matter.

We downloaded the training PAC2019 data consisting of 2,640 unique participants, from which we kept a subset of 533 (20%) selected by random sampling to be representative of the full training sample (in terms of age, sex, and site origin, Supplementary Table 8). We used those 533 participants to benchmark the prediction accuracy of each of our models (paired *t*-test), as well as to find the optimal weights when combining the different predicted ages (Figure 5).

On the 2,107 images left in the training set, we performed a 5-fold cross-validation to estimate the C and gamma hyperparameters in SVM. For the deep learning algorithms, the best epoch was determined using an 80:20 train–validation split, the same for all algorithms. Note that BLUP does not require hyperparameter estimation and was trained on the 2,107 images (see Figure 5 for summary).

#### Model Combination

We estimated the optimal combination of age predictors using a linear regression of all predictions on self-reported age, in the sample of 533 participants. The regression coefficients were then applied in the PAC2019 test sample to weight the different scores (Figure 5).

To evaluate the improvement in MAE resulting from ensemble learning, we randomly split the 533 hold-out sample in halves. We trained the linear model on the first half and evaluated the MAE on the other half, which provides an unbiased estimate of the variance of the MAE. Because the 50:50 split was performed at random (with no guarantees that sex, age, and site distributions were representative), we iterated this process 500 times and report the bootstrap estimate of the standard error (SE). Similarly, we tested whether the MAE of ensemble learning was significantly lower than that of the best algorithm.

#### Reducing Bias—Predictive Analytics Competition 2019 Challenge 2

We observed that scaling each age score using the median and mean absolute deviation greatly reduced the bias, as defined by the Spearman correlation between age and prediction error. Thus, we transformed each score and combined them using linear regression as described above (Figure 5).

### Additional Experiments

#### Individual Performance of Each Algorithm

We used a 5-fold cross-validation design on the 2,640 PAC individuals, to evaluate the stability of the prediction accuracy of each algorithm. Folds were selected to be representative of the full sample (Supplementary Table 3). Note that the split performed for the PAC challenge (see *Training and Validation Procedures*) is the first fold of the cross-validation. We used paired *t*-tests to compare the performance of the algorithms.

#### Different Types of Model Combination: Linear Regression vs. Random Forest

Ensemble methods combine several algorithms into one single algorithm and are powerful techniques to improve predictive performance (53). We explored different types of combinations: (i) mean score for each individual; (ii) median score; (iii) linear combination with weights estimated from linear regression; and (iv) scores combination from random forest regression.

For linear regression and random forest, we trained the ensemble algorithms on a random subset comprising half of the hold-out sample (N ~ 265) and calculated the MAE on the other half. We repeated this process 500 times to get a bootstrap estimate of the SE of the MAE, as well as to test differences between ensemble learning and our best single algorithm. The random forest regression was composed of 100 trees of maximum depth 15 and was trained so as to minimize the MAE.

#### Combining Seven (Identical) Convolutional Neural Networks or the Seven Best Epochs

We wanted to compare the ensemble prediction accuracy achieved from our seven algorithms with the accuracy that may be achieved from combining several predictions from the same CNN architecture as well as from the seven best epochs of a single CNN. We wanted to answer the question “Is ensemble learning accuracy driven by the sheer number of scores combined?” and its correlate “Is it better to combine different algorithms with different underlying architectures and prediction error type?” We chose to focus on the Inception V1 CNN, as it minimized the MAE among the algorithms considered.

#### Influence of the Type of Brain Features on Prediction Accuracy

We investigated the impact of the input features by training the BLUP and SVM models on the gray matter maps, in replacement of the vertex-wise surfaces used previously. We did not train the deep learning algorithms on surface-based processed images, as it is difficult to integrate the spatial relationship of the vertices that compose a 2D surface folded into gyri and sulci. In addition, we evaluated the impact of replacing BLUP and SVM by their gray matter maps equivalent in ensemble learning, using linear combination.

#### Sex, Age, and Site Bias

We studied the impact of sex, age, and site on the error (and absolute error) of each algorithm trained the first fold of the cross-validation design. We used, for each algorithm, a linear mixed effect model, modeling age of the participants as a fixed effect and sex and site as random effects. The impact of each effect was evaluated using a log-likelihood ratio test. We used Bonferroni correction to account for multiple comparisons.

## Results

### Predictive Analytics Competition Challenge Results

For the first challenge of minimizing the MAE, the deep learning models performed significantly better than BLUP or SVM (*p*-value <3.1E−4, paired *t*-test) with a MAE between 3.82 (Inception) and 4.18 years (six-layer CNN, Table 1), compared with a MAE >4.90 years (BLUP-quantiles, Table 1). Performance of the deep learning algorithms was not significantly different from each other when accounting for multiple testing (*p* > 0.027). All the models returned biased predictions with rank correlations between age and prediction error >0.24 (Table 1). Ensemble prediction yielded lower MAE estimates (3.46 years, Table 1), which represented a significant improvement over the Inception performance (mean improvement 0.36 years, SE = 0.099 [bootstrap], paired *t*-test *p* = 1.3E−4). The performance observed on the independent PAC sample (3.33 years, third best prediction) aligned with our ensemble prediction estimate.

**Table 1:** Mean absolute error (standard error) and Spearman correlation coefficient (ρ) between age and prediction error for each model on the validation set.

		**BLUP-mean**	**BLUP-quantiles**	**SVM**	**6-layer CNN**	**Age spe. 6-layer CNN**	**ResNet**	**Inception V1**	**Ensemble prediction**	**PAC results**
First challenge	MAE (SE)	5.32 (0.19)	4.90 (0.19)	5.31 (0.18)	4.18 (0.16)	4.01 (0.15)	4.02 (0.15)	3.82 (0.14)	3.46 (0.13)^*^	3.33
	|ρ|	0.32	0.37	0.58	0.25	0.30	0.24	0.41	0.32	0.21
Second challenge	MAE (SE)	6.15 (0.23)	5.96 (0.23)	6.14 (0.23)	5.27 (0.21)	5.17 (0.20)	5.25 (0.20)	4.97 (0.19)	4.69 (0.19)^*^	4.83
	|ρ|	0.14	0.15	0.15	0.084	0.068	0.11	0.058	0.058	0.021

The standard error [SE = SD/sqrt(N)] reflects the uncertainty around the MAE estimate. A 95% confidence interval may be calculated as MAE ± 1.96*SE, though it (falsely) assumes normality of the absolute error distribution. We performed ensemble prediction using linear combination of age predictors, with linear weights estimated via linear regression. SE of the MAE for ensemble prediction were calculated by bootstrap.

* *Indicates a significant reduction of MAE via ensemble learning compared with Inception alone (p <0.05). PAC results were provided by the PAC team and estimated on participants not available to the authors*.

For the second challenge, we rescaled the predictions using the median and the mean absolute deviation per site. This resulted in an increased MAE of about 1 year but substantially decreased the bias (Table 1). Again, ensemble learning resulted in a significant improvement of the performance over that of Inception (mean improvement 0.30, SE = 0.13, *p* = 0.010). We achieved a MAE of 4.83 years in the PAC test sample with a bias of ρ = 0.021 (sixth best performance from six entries).

### Additional Experiments

#### Effect of Train/Test Split

We sought to evaluate whether our conclusions were dependent on the train/test split used in the previous section by performing a 5-fold cross-validation experiment. In each fold, we found nominal significant differences in MAE between BLUP/SVM and ResNet (paired *t*-test, *p* < 5.5E−3) (Table 2). The difference between BLUP/SVM and Inception V1 was significant in four of the folds (*p* < 5.3E−5). Results were a lot more contrasted for the differences between BLUP/SVM and the six-layer CNNs that were significant in only 2- or 3-folds. Bias was greater than the PAC threshold for challenge 2 (0.10) for all scores and folds (ρ ranging from 0.15 to 0.53, Supplementary Table 9).

**Table 2:** Mean absolute error (standard error) for each model and each fold (first challenge).

				**Individual algorithms**				**Ensemble learning**			
	**BLUP-mean**	**BLUP-quantiles**	**SVM**	**6-layer CNN**	**Age spe. 6-layer CNN**	**ResNet**	**Inception V1**	**LM**	**RF**	**Mean**	**Median**
Fold 1	5.32 (0.19)	4.90 (0.19)	5.31 (0.18)	4.18 (0.16)	4.01 (0.15)	4.02 (0.15)	3.82 (0.14)	3.46 (0.13)^*^	3.62 (0.15)	3.74 (0.13)	3.67 (0.14)
Fold 2	5.05 (0.18)	4.79 (0.19)	5.34 (0.18)	4.47 (0.15)	4.12 (0.13)	4.01 (0.14)	3.97 (0.15)	3.53 (0.13)^*^	3.60 (0.15)^*^	3.69 (0.13)	3.74 (0.13)
Fold 3	4.90 (0.18)	4.37 (0.16)	4.84 (0.17)	4.41 (0.16)	4.27 (0.15)	3.88 (0.14)	4.00 (0.16)	3.33 (0.13)^*^	3.46 (0.15)^*^	3.46 (0.12)^*^	3.45 (0.13)^*^
Fold 4	5.07 (0.18)	4.71 (0.18)	5.06 (0.18)	4.55 (0.17)	4.27 (0.16)	4.11 (0.15)	3.85 (0.15)	3.57 (0.13)^*^	3.72 (0.14)	3.68 (0.14)	3.74 (0.15)
Fold 5	5.22 (0.19)	4.69 (0.18)	5.20 (0.18)	4.02 (0.16)	3.89 (0.15)	3.99 (0.16)	3.75 (0.15)	3.34 (0.13)^*^	3.51 (0.14)	3.56 (0.13)	3.47 (0.13)
5-fold combined MAE	5.11	4.69	5.15	4.33	4.11	4.00	3.88	3.44	3.58	3.62	3.61

Fold 1 corresponds to the train-test split used in the Predictive Analytics Competition (PAC) challenge and presented in Table 1. LM (linear model), RF (random forest), mean, and median age scores are the four methods considered for ensemble learning. The standard error [SE = SD/sqrt(N)] reflects the uncertainty around the MAE estimate. A 95% confidence interval may be calculated as MAE ± 1.96 * SE, though it (falsely) assumes normality of the absolute error distribution. For the 5-fold combined MAE, we did not report the SE, as it is notoriously biased downward (54) due to the overlap of the different training/test samples.

* *Indicates a significant reduction of MAE via ensemble learning compared with Inception alone (p < 0.01, assuming five independent tests)*.

#### Strategies for Model Combination

In each of the 5-folds, the combined age score using linear regression outperformed the prediction from Inception V1 (*p* < 0.0022). Ensemble learning via random trees was significantly better than Inception V1 alone for folds 2 and 3 only (*p* = 4.0E−3 and 3.4E−4). To note, the MAE achieved with random forest was very close to the MAE obtained by taking the average or median scores for each individual (Table 2). We could not conclude about a significant difference between linear model combination and random forest (*p* > 0.035).

When rescaling scores for the second challenge, we observed a consistent increase in MAE, for all algorithms and folds (Supplementary Table 10), though the bias was greatly reduced and met the PAC challenge criteria (ρ <0.10) in most cases (Supplementary Table 11). Ensemble learning with linear regression significantly improved the MAE in four of the folds (*p* < 0.0038) and satisfied the low bias criteria in all cases (ρ <0.058, Supplementary Tables 10, 11). On the other hand, random forest combination greatly reduced the MAE, compared with linear combination (*p* < 1E−5), but always exceeded the low bias threshold (ρ > 0.34, Supplementary Table 11).

Since linear model combination of scores appeared to minimize MAE and preserve low bias, we plotted the linear weights attributed to each algorithm, for each fold and bootstrap iteration (Supplementary Figures 1, 2). We observed highly variable weighting, dependent on the folds, as well as on the later splits on which the linear coefficients were estimated. To note, no algorithm consistently received a null weight that would be suggestive of no contribution to the ensemble learning.

#### Ensemble Learning From Seven Inception V1, Seven Best Epochs, and From All Age Scores

Instead of combining seven different algorithms, we evaluated the combination (using linear regression) of seven Inception V1 algorithms, as well as the seven best epochs of a single Inception V1 optimization. Due to the computing resources needed to optimize a deep learning algorithm, we only performed this experiment on the first train/test fold (used in Table 1 for example).

The seven best epochs individually achieved MAE in the range of 3.68–4.27, while the seven Inception V1 models predicted age with a MAE between 3.52 and 3.89. Combining seven epochs resulted in a MAE of 3.71 (SE = 0.13), while combining seven Inception V1 achieved a MAE of 3.46 (SE = 0.13), which was comparable with the performance obtained by combining seven different algorithms (Table 1).

Further combining all scores (seven epochs, seven Inception V1, and seven original scores) only resulted in a marginal improvement of the age prediction: MAE = 3.41 (SE = 0.14, *p* > 0.05).

#### Choice of the Type of Features

The lower performance of BLUP/SVM compared with deep learning algorithms led us to test whether it could be attributed to the input data, or the algorithms themselves. Thus, we retrained BLUP and SVM on the same gray matter maps used by all the deep learning algorithms. We found that for two of the folds, BLUP-mean and SVM trained on gray matter maps resulted in improved prediction, compared with the surface trained equivalents. The improvement of BLUP-quantiles was significant in three of the 5-folds (Table 3).

**Table 3:** Mean absolute error (standard error) for the best linear unbiased predictor (BLUP) and support vector machine (SVM) models trained on gray matter maps for each fold.

	**BLUP-mean**	**BLUP-quantiles**	**SVM**	**Ensemble learning**
Fold 1	4.51 (0.16)^†^^*^	3.91 (0.14)^†^	4.64 (0.17)^†^^*^	3.39 (0.13)
Fold 2	4.45 (0.16)^†^^*^	4.06 (0.15)^†^	4.75 (0.16)^†^^*^	3.46 (0.13)
Fold 3	4.67 (0.17)^*^	4.02 (0.16)	4.62 (0.17)^*^	3.26 (0.13)
Fold 4	4.59 (0.16)^*^	4.16 (0.16)^†^	4.52 (0.16)^*^	3.55 (0.14)
Fold 5	4.86 (0.18)^*^	4.21 (0.17)	4.78 (0.17)^*^	3.35 (0.14)
5-fold MAE	4.61	4.07	4.66	3.42

The standard error [SE = SD/sqrt(N)] reflects the uncertainty around the mean absolute error (MAE) estimate. A 95% confidence interval may be calculated as MAE ± 1.96 *SE, though it (falsely) assumes normality of the absolute error distribution. For the 5-fold combined MAE, we did not report the SE, as it is notoriously biased downward (54) due to the overlap of the different training/test samples.

† *Algorithm trained on gray matter maps performs significantly better than the same algorithm trained on surface-based vertices (p <0.05/15)*.

* *Algorithm trained on gray matter maps performs significantly worse than Inception V1 (p < 0.05/15). Ensemble learning was performed using linear regression and included the seven algorithms considered in Tables 1, 2, in addition to the three introduced in this section*.

Despite the reduction in MAE, BLUP-mean and SVM trained on gray matter maps still performed worse than Inception V1 (*p* < 0.0033, Table 3), though the difference between Inception V1 and BLUP-quantiles became non-significant in all folds (Table 3).

Including the gray matter map-based BLUP and SVM predictions did not improve the performance of ensemble learning over what has been reported above (Tables 2, 3).

#### Sex, Age, and Site Association With Prediction Error

Age correlated positively with prediction error (calculated as age – predicted age) for all algorithms in the first train/test split (Table 4). Thus, predicted age tended to underestimate the age of older participants and overestimate age of younger individuals. Such results align with the large rank bias reported in Tables 1, 2. We did not observe a significant association of prediction error with sex or site (Table 4).

**Table 4:** p-values for the effect of age, site, and sex on prediction error for the seven models on fold 1.

	**BLUP-mean**	**BLUP-quantiles**	**SVM**	**6-layer CNN**	**Age spe. 6-layer CNN**	**ResNet**	**Inception V1**
Age	2.9E−10^*^	5.8E−13^*^	5.8E−46^*^	7.3E−10^*^	2.2E−13^*^	9.1E−05^*^	7.7E−20^*^
Site	3.7E−01	4.4E−02	4.5E−03	2.8E−02	4.3E−02	2.3E−02	5.0E−02
Sex	7.1E−02	1.4E−01	3.6E−02	1.0E+00	8.5E−01	1.0E+00	5.4E−01

* *Significant after correction for multiple comparisons (i.e., p < 0.05/21 or p < 2.3E−3)*.

We found the same pattern of association with absolute error (*p* < 1.8E−4 with age), suggesting that older participants contributed most to the MAE (Table 4).

#### Morphometricity of Age as Upper Bound of Prediction Accuracy

From BLUP models, we estimated the total association between age and the brain features. Morphometricity is expressed in proportion of the variance (*R*^2^) of age; thus, it quantifies how much of the differences in age in the sample may be attributed/associated with variation in brain structure. With surface-based processing (~650,000 vertices), we estimated the morphometricity to be *R*^2^ = 0.99 (SE = 0.052), while for volume-based processing (~480,000 voxels), it reached *R*^2^ = 0.97 (SE = 0.015).

## Discussion

Here, we describe the ensemble learning of seven different age predictions from T1w MRI images, which led to a MAE of 3.33 years on an independent dataset, held by the organizers of the PAC2019. From all worldwide competitors, our prediction ranked third, though we only narrowly beat teams ranking fourth (MAE = 3.33) and fifth (MAE = 3.37). To note, the gap was more consequent with teams who ranked first (MAE = 2.90), second (MAE = 3.09), or sixth (MAE = 3.55). In absence of reported SE in the PAC results, we cannot conclude whether the different prediction accuracies are statistically different from each other. It is important to keep in mind that ranking of prediction accuracy may be highly dependent on the metric chosen as well as on the test data (55). Statistical testing can provide a confident ranking of algorithms, and inclusion of other datasets is needed to conclude about the generalizability (and performance) of the prediction scores on samples with other demographics, MRI machines, or patient groups for instance.

In this publication, we sought to detail our approach, facilitate replication, and reuse of our code/results and also to identify factors influencing the prediction accuracy we achieved. We present analyses that we performed prior to the challenge closing (that informed our method), as well as *post-hoc* analyses in which we explored new avenues. More precisely, we evaluated the effect on performance of (i) algorithm choice, (ii) ensemble learning methods, (iii) feature input/data processing, (iv) number and type of scores in ensemble learning, and (v) covariates such as age, sex, and site. Lastly, we detail our approach for the second PAC challenge (minimize MAE, while controlling bias) though in much less detail as we came sixth (out of six entries) with a MAE almost 2 years greater than the winner.

We found that the four deep learning algorithms (ResNet, Inception V1, and custom six-layer CNN) outperformed (by almost 1 year of MAE) simpler algorithms (BLUP and SVM) in most train/test splits considered (Tables 1, 2), with the exception of BLUP-quantiles trained on gray matter maps. We could not conclude about a significant difference between the performance of deep learning algorithms, though the size of our test sample (~530) limited our statistical power to detect small differences.

Ensemble learning with weights estimated via linear regression led to a significant reduction of MAE of about 0.4 years (Table 2). Score combination using random forest also outperformed the algorithm with minimal MAE (Inception V1), but the result was somewhat dependent on the folds considered. The difference between linear model and random forest was too small to conclude about a significant difference (Table 2). The weights given to each algorithm via linear regression were highly dependent on the folds and iterations, which might be an artifact of the large correlations between the scores. Nevertheless, few weights were consistently set to 0 (across all folds and iterations), suggesting that all seven algorithms contributed to the ensemble learning (Supplementary Figures 1, 2). Our results align with previous publications that highlighted the benefits of ensemble learning, which combines different models (56) or different data (57).

BLUP and SVM performed better (~0.7 years' progression in MAE) when trained on gray matter maps (voxel-based morphometry in gray matter) compared with surface-based features (vertex-wise measurements of gray matter thickness and surface area). Despite the improvement, the performance of BLUP-mean and SVM was still significantly lower than that of Inception V1. To note, the difference between BLUP-quantiles and Inception V1 became non-significant. Here, we compared two competing approaches of processing T1w MRIs, implemented in two software suites [FreeSurfer (25) and SPM]. Each processing stream allows multiple user-defined options (e.g., on registration, normalization, and templates) whose effect on age prediction is not known. Importantly, the image processing maximizing age prediction may not be the best suited to predict another phenotype (e.g., disease status). Lastly, the good performance of BLUP-median on gray matter maps raises the question of cost-efficiency and updatability of prediction, considering that deep learning models require about 24 h of computing on a GPU, while BLUP only takes a few minutes on a single CPU.

In addition, we found very similar performance of ensemble prediction from our seven different algorithms compared with that of seven independently trained Inception V1 scores. We conclude that using a variety of algorithms may not offer an advantage over using several (well-performing) ones. Due to limited computing resources, we did not investigate whether increasing the number of Inception V1 algorithms further reduced the MAE, though our age prediction did not progress when combining the 21 models estimated throughout the analysis.

Finally, our predictions showed a large age bias: overestimating age on younger participants and underestimating it on older participants. We also identified older individuals as main contributors of the MAE, suggesting much is to be gained by improving the performance on this sub-population. Our attempt to re-train part of the network on adults above 40 years of age (age specialized six-layer CNN) was not conclusive in improving the age prediction accuracy. Other avenues for research include enriching the training sample in specific age groups or demographics that show a lower performance. We did not find error or absolute error to be associated with sex or site, despite differences in global head size, or site differences in term of scanners, demographics, and image qualities. An investigation on a larger dataset may be more powered in detecting subgroups with larger MAE. To finish on bias, we found that rescaling the scores using the median and median absolute deviation (per site) could reduce drastically the bias but resulted in an increase in MAE (Table 1 and Supplementary Tables 10, 11). Low bias age predictors avoid subsequent association analyses (e.g., in case–control samples) to be confounded by age, though it may be safer to always control for age in PAD analyses (15).

We did not systematically investigate the use of white matter maps to improve prediction accuracy. Only the six-layer CNN was trained on both gray matter and white matter maps, and it did not outperform the other algorithms. In addition, our 80:20 split design allowed for (well-powered) statistical testing and weighted estimation for ensemble learning; however, it may not be the optimal split to minimize the MAE. Overall, we estimated the theoretical upper bound of linear prediction to be *R*^2^ = 0.97 (SE = 0.015), though we do not know the corresponding MAE. In comparison, our best BLUP score (Table 3) achieved *R*^2^ = 0.94, and the ensemble learning model that minimized the MAE (Table 1) achieved a prediction *R*^2^ of 0.96. This suggests that the prediction accuracy we report here might be close to the theoretical maximum achievable from linear predictors, even though this claim is weakened by the fact that prediction *R*^2^ is not a sufficient statistic here as age was not normally distributed (thus, it might be inflated). Importantly, the high prediction accuracy we report does not ensure that PAD best discriminates cases from controls in a clinical sample (23).

More generally, prediction accuracy is not a linear function of training sample size [see (58)], and we can expect further significant improvement in age prediction to require much larger sample sizes. We would also like to point out that reducing the MAE below 1 year is unlikely, when training algorithms on rounded age, which was the case here. Finally, PAC participants were described as healthy individuals, though screening of all brain related disorders is impossible, which raises the question of unknown diagnosis for participants with large prediction error.

In conclusion, we achieved a MAE of 3.33 years to predict age from T1w MRI. We identified several contributors to prediction accuracy: algorithm choices, image processing options, and ensemble learning.

## URLs

ENIGMA protocol for subcortical processing: http://enigma.ini.usc.edu/protocols/imaging-protocols/.

OSCA software: http://cnsgenomics.com/software/osca/#Overview.

## Data Availability Statement

Publicly available datasets were analyzed in this study. The ensemble of datasets presented in this article are not readily available because they are held and distributed by the PAC2019 team. Requests to access the datasets should be directed to Pr. Hahn (hahnt@wwu.de) and Pr. Cole (james.cole@imperial.ac.uk).

## Ethics Statement

Ethical review and approval was not required for the study on human participants in accordance with the local legislation and institutional requirements. The patients/participants provided their written informed consent to participate in this study.

## Author Contributions

BC-D, JF, BM, ET-S, and AW had full access to all the data in the study and take responsibility for the integrity of the data and the accuracy of the data analysis. Study concepts and study design were provided by BC-D, JF, BM, ET-S, AW, MA, NB, and OC. Acquisition, analysis, or interpretation of data was performed by all authors. Manuscript drafting or manuscript revision for important intellectual content was performed by all authors. Approval of final version of submitted manuscript was done by all authors. Literature research was performed by BC-D, JF, BM, ET-S, AW, and NB. Statistical analysis was performed by BC-D, JF, BM, ET-S, AW, and MA. Funding was obtained by SD, DD, NB, and OC. Administrative, technical, or material support was provided by SD, DD, NB, and OC. Study supervision was carried out by NB and OC.

## Conflict of Interest

OC reports having received consulting fees from AskBio (2020), fees for writing a lay audience short paper from Expression Santé (2019), and speaker fees for a lay audience presentation from Palais de la découverte (2017) and reports that his laboratory has received grants (paid to the institution) from Air Liquide Medical Systems (2011–2016) and Qynapse (2017–present). The members from his laboratory have co-supervised a Ph.D. thesis with myBrainTechnologies (2016-present). OC's spouse is an employee of myBrainTechnologies (2015–present). OC and SD have submitted a patent to the International Bureau of the World Intellectual Property Organization (PCT/IB2016/0526993, Schiratti J-B, Allassonniere S, OC, SD, a method for determining the temporal progression of a biological phenomenon and associated methods and devices) (2016). The remaining authors declare that the research was conducted in the absence of any commercial or financial relationships that could be construed as a potential conflict of interest.
