# Supplementary material for: Ensemble Learning of Convolutional Neural Network, Support Vector Machine, and Best Linear Unbiased Predictor for Brain Age Prediction: ARAMIS Contribution to the Predictive Analytics Competition 2019 Challenge
Source: Front Psychiatry. 2020 Dec 15;11:593336. doi: 10.3389/fpsyt.2020.593336 (PMC7770104; doi:10.3389/fpsyt.2020.593336)
Supplement: Supplementary file 1 [file Data_Sheet_1.pdf]

Supplementary Material

Supplementary Table 1: 6-layer CNN architecture

The padding size in max pooling layers depends on the input: columns of zeros are added along a dimension until the size along this dimension is a multiple of the stride size. A ReLU activation is used for the model.

layer	output size	kernel size	stride	padding	dilation
input	1 × 121 × 145 × 121				
convolution	8 × 121 × 145 × 121	3	1	1	1
batch normalization					
activation					
max pooling	8 × 61 × 73 × 61	2	2	*	1
convolution	16 × 61 × 73 × 61	3	1	1	1
batch normalization					
activation					
max pooling	16 × 31 × 37 × 31	2	2	*	1
convolution	32 × 31 × 37 × 31	3	1	1	1
batch normalization					
activation					
max pooling	32 × 16 × 19 × 16	2	2	*	1
convolution	64 × 16 × 19 × 16	3	1	1	1
batch normalization					
activation					
max pooling	64 × 8 × 10 × 8	2	2	*	1
convolution	128 × 8 × 10 × 8	3	1	1	1
batch normalization					
activation					
max pooling	128 × 4 × 5 × 4	2	2	*	1
dropout (80%)	10 240				
linear	1				

Supplementary Table 3: Resnet architecture

An ELU activation function is used for this model.

layer name	layer type	output size	kernel size	stride	padding	dilation
input		1 × 121 × 145 × 121				
residual block 1	convolution	1 × 121 × 145 × 121	3	1	1	1
	batch normalization					
	activation					
	convolution		3	1	1	1
	batch normalization					
	convolution shortcut		1	1	0	1
	concatenation					
	activation					
max pooling		8 × 60 × 72 × 60	3	2	0	1
residual block 2	convolution	8 × 60 × 72 × 60	3	1	1	1
	batch normalization					
	activation					
	convolution		3	1	1	1
	batch normalization					
	convolution shortcut		1	1	0	1
	concatenation					
	activation					
max pooling		16 × 29 × 35 × 29	3	2	0	1
residual block 3	convolution	16 × 29 × 35 × 29	3	1	1	1
	batch normalization					
	activation					
	convolution		3	1	1	1
	batch normalization					
	convolution shortcut		1	1	0	1
	concatenation					
	activation					
max pooling		32 × 14 × 17 × 14	3	2	0	1
residual block 4	convolution	32 × 14 × 17 × 14	3	1	1	1
	batch normalization					
	activation					
	convolution		3	1	1	1
	batch normalization					
	convolution shortcut		1	1	0	1
	concatenation					
	activation					
max pooling		64 × 6 × 8 × 6	3	2	0	1
residual block 5	convolution	64 × 6 × 8 × 6	3	1	1	1
	batch normalization					
	activation					
	convolution		3	1	1	1

	batch normalization					
	convolution shortcut		1	1	0	1
	concatenation					
	activation					
max pooling		128 × 2 × 3 × 2	3	2	0	1

Supplementary Table 4: Resnet fully connected layer

layer name	output size
input	128 × 2 × 3 × 2
linear	256
activation	256
dropout (80%)	256
add covariable	256 + 2
linear	1

Supplementary Table 5: 3D-Inception-v1 architecture - Stem Network

A ReLU activation is used for the model.

**Layer**	**output size**	**kernel size**	**stride**	**padding**	**dilation**
input	1 × 121 × 145 × 121				
convolution	64 × 59 × 71 × 59	7	2	1	1
batch normalization					
activation					
max pooling	64 × 29 × 35 × 29	3	2		
convolution	64 × 29 × 35 × 29	1	1	1	1
batch normalization					
activation					
convolution	129 × 29 × 35 × 29	3	2	1	1
batch normalization					
activation					
max pooling	129 × 14 × 17 × 14	3	2		

Supplementary Table 6: 3D-Inception-v1 architecture - Auxiliary Regression

The padding size in max pooling layers depends on the input: columns of zeros are added along a dimension until the size along this dimension is a multiple of the stride size. A ReLU activation is used for the model.

**layer**	**output size**	**kernel size**	**stride**	**padding**	**dilation**
inception (4a | 4d)	(512|528) × 6 × 8 × 6				
average pooling	(512|528) × 3 × 3 × 3	3			
convolution	128 × 3 × 3 × 3	1	1	1	1
batch normalization					
activation					
resizing	3456				
linear	1024				
dropout (70%)	1024				
linear	1				

Supplementary Table 7: 3D-Inception-v1 architecture - Main Architecture

The padding size in max pooling layers depends on the input: columns of zeros are added along a dimension until the size along this dimension is a multiple of the stride size. A ReLU activation is used for the model.

layer	output size	kernel size	stride	#1x1	#3x3red	#3x3	#3x3red(2)	#3x3(2)	Pool proj
stem network	192 × 14 × 17 × 14								
inception (3a)	256 × 14 × 17 × 14			64	96	128	16	32	32
inception (3b)	480 × 14 × 17 × 14			128	128	192	32	96	64
max pooling	480 × 6 × 8 × 6	3	2						
inception (4a)	512 × 6 × 8 × 6			192	96	208	16	48	64
inception (4b)	512 × 6 × 8 × 6			160	112	224	24	64	64
inception (4c)	512 × 6 × 8 × 6			128	128	256	24	64	64
inception (4d)	528 × 6 × 8 × 6			112	144	288	32	64	64
inception (4e)	832 × 6 × 8 × 6			256	160	320	32	128	128
max pooling	832 × 2 × 3 × 2	3	2						
inception (5a)	832 × 2 × 3 × 2			256	160	320	32	128	128
inception (5b)	1024 × 2 × 3 × 2			384	192	384	48	128	128
average pooling	1024 × 1 × 1 × 1	2 × 3 × 2	1						
dropout (70%)	1024 × 1 × 1 × 1								
resizing	1024								
linear	1								

Supplementary Table 8: Demographics of the complete PAC training sample and of each test fold

	age	sex
	Mean (SD)	% females
Fold 1	35.8 (16.6)	56
Fold 2	35.9 (16.2)	54
Fold 3	36.0 (16.1)	51
Fold 4	35.9 (16.2)	53
Fold 5	35.9 (15.9)	51
Total sample	35.9 (16.2)	53

Supplementary Table 9: Spearman correlation between PAD and chronological age for each model and each fold for the first challenge.

	**BLUP mean**	**BLUP quantiles**	**SVM**	**6-layers CNN**	**Age spe. 6-layers CNN**	**ResNet**	**Inception V1**	**Ensemble learning**			
								**LM**	**RT**	**mean**	**median**
**Fold 1**	0.32	0.37	0.58	0.25	0.30	0.24	0.41	0.32	0.39	0.43	0.42
**Fold 2**	0.25	0.29	0.52	0.19	0.28	0.29	0.32	0.33	0.41	0.37	0.37
**Fold 3**	0.18	0.18	0.53	0.42	0.39	0.32	0.28	0.32	0.36	0.42	0.39
**Fold 4**	0.22	0.23	0.47	0.47	0.46	0.37	0.39	0.35	0.38	0.46	0.45
**Fold 5**	0.23	0.23	0.52	0.15	0.24	0.32	0.38	0.25	0.36	0.37	0.37

Supplementary Table 10: Mean absolute error (standard error) for each model and each fold (second challenge).

Fold 1 corresponds to the train-test split used in the PAC challenge and presented in Table 1. LM (linear model), RF (random forest), mean and median age scores are the four methods considered for ensemble learning. The standard error (SE = SD/sqrt(N)) reflects the uncertainty around the MAE estimate. A 95% confidence interval may be calculated as MAE ± 1.96*SE, though it (falsely) assumes normality of the absolute error distribution. For the 5-fold combined MAE we did not report the SE as it is notoriously biased downward (Bengio & Grandvalet, 2004) due to the overlap of the different training/test samples. * indicates a significant reduction of MAE via ensemble learning compared to Inception alone (p<0.01, assuming 5 independent tests).

	**BLUP mean**	**BLUP quantiles**	**SVM**	**6-layer CNN**	**Age spe. 6-layer CNN**	**ResNet**	**Inception V1**	**Ensemble learning**			
								**LM**	**RF**	**mean**	**median**
**Fold 1**	6.56 (0.25)	6.78 (0.26)	6.62 (0.25)	6.13 (0.24)	6.10 (0.23)	6.38 (0.23)	5.92 (0.22)	4.69 (0.19)	3.82 (0.15)	4.77 (0.19)	4.90 (0.19)
**Fold 2**	8.39 (0.32)	7.93 (0.31)	7.88 (0.30)	8.03 (0.31)	7.76 (0.29)	7.68 (0.30)	7.74 (0.30)	5.30 (0.19)	4.14 (0.19)	5.32 (0.19)	5.34 (0.20)
**Fold 3**	7.17 (0.27)	7.27 (0.27)	6.94 (0.26)	6.95 (0.26)	7.02 (0.26)	6.96 (0.25)	6.91 (0.26)	5.00 (0.20)	3.97 (0.16)	4.94 (0.19)	5.17 (0.20)
**Fold 4**	7.19 (0.28)	7.35 (0.28)	7.10 (0.28)	6.79 (0.27)	6.80 (0.26)	6.89 (0.27)	6.76 (0.26)	4.95 (0.19)	4.13 (0.16)	4.91 (0.18)	5.07 (0.19)
**Fold 5**	8.22 (0.35)	7.74 (0.31)	7.32 (0.30)	7.06 (0.28)	6.87 (0.27)	7.31 (0.29)	7.01 (0.26)	5.42 (0.22)	4.06 (0.17)	5.28 (0.23)	5.26 (0.23)
**5-fold combined**	7.50	7.41	7.17	6.99	6.91	7.04	6.87	5.07	4.02	5.04	5.15

Supplementary Table 11: Spearman correlation between brain PAD and chronological age for each model on each fold for the second challenge.

Cells in bold correspond to correlation > 0.10, which was the maximal bias allowed to enter the second PAC2019 challenge.

	**BLUP mean**	**BLUP quantiles**	**SVM**	**6-layer CNN**	**Age spe. 6-layer CNN**	**ResNet**	**Inception V1**	**Ensemble learning**			
								**LM**	**RF**	**mean**	**median**
**Fold 1**	**0.14**	**0.15**	**0.15**	0.085	0.068	**0.11**	0.058	0.058	**0.40**	**0.13**	**0.11**
**Fold 2**	0.023	0.026	0.018	0.008	0.057	0.012	0.026	0.042	**0.38**	0.0068	0.013
**Fold 3**	0.013	0.016	0.016	0.005	0.033	0.024	0.035	0.045	**0.34**	0.013	0.0089
**Fold 4**	0.066	0.042	0.064	0.08	0.052	0.01	0.024	0.014	**0.41**	0.056	0.048
**Fold 5**	0.046	0.032	0.035	0.004	0.043	0.014	0.011	0.037	**0.40**	0.024	0.026

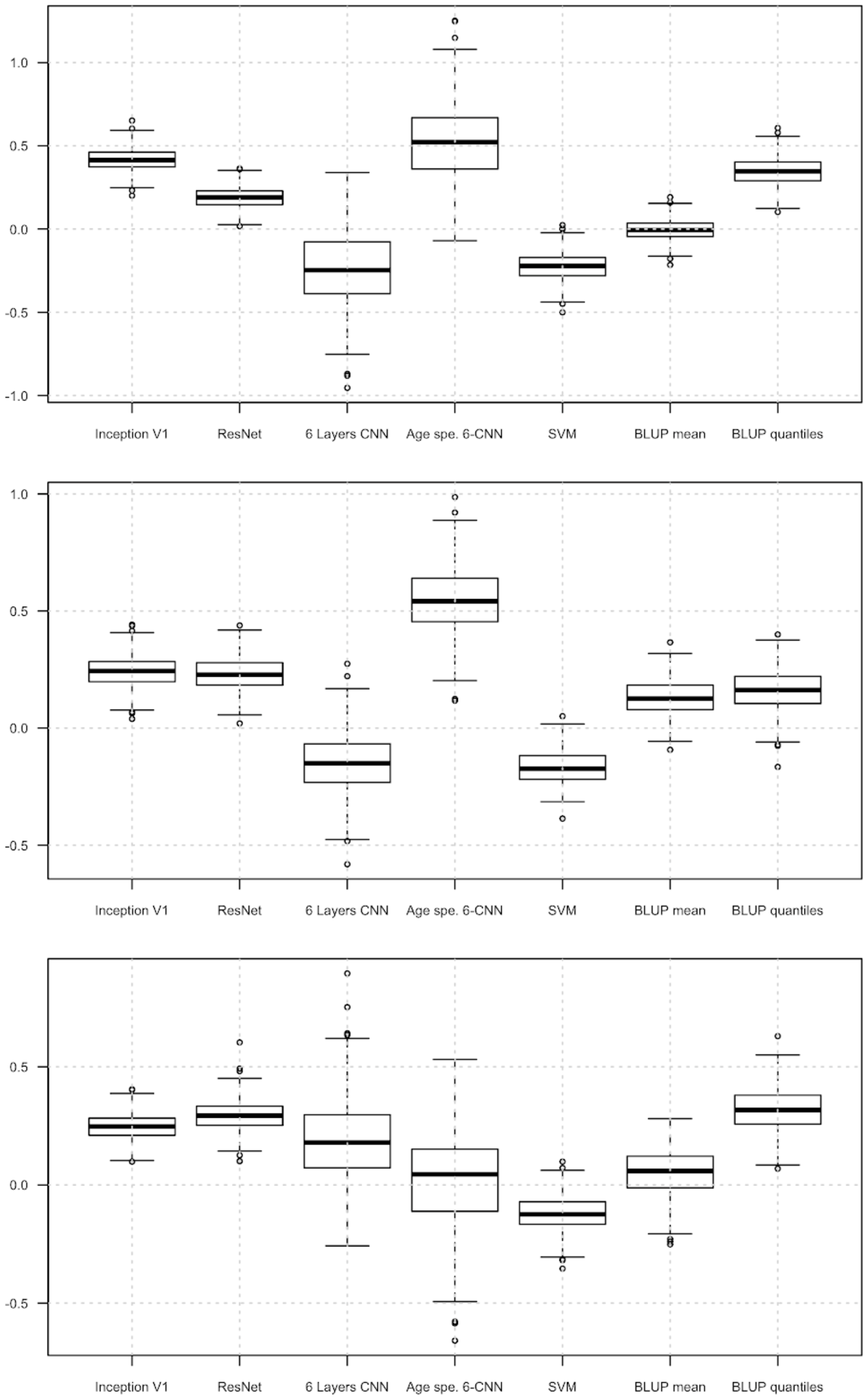
1.0
0.5
0.0
-0.5
-1.0
Inception V1
ResNet
6 Layers CNN
Age spe. 6-CNN
SVM
BLUP mean
BLUP quantiles
1.0
0.5
0.0
-0.5
Inception V1
ResNet
6 Layers CNN
Age spe. 6-CNN
SVM
BLUP mean
BLUP quantiles
0.5
0.0
-0.5
Inception V1
ResNet
6 Layers CNN
Age spe. 6-CNN
SVM
BLUP mean
BLUP quantiles

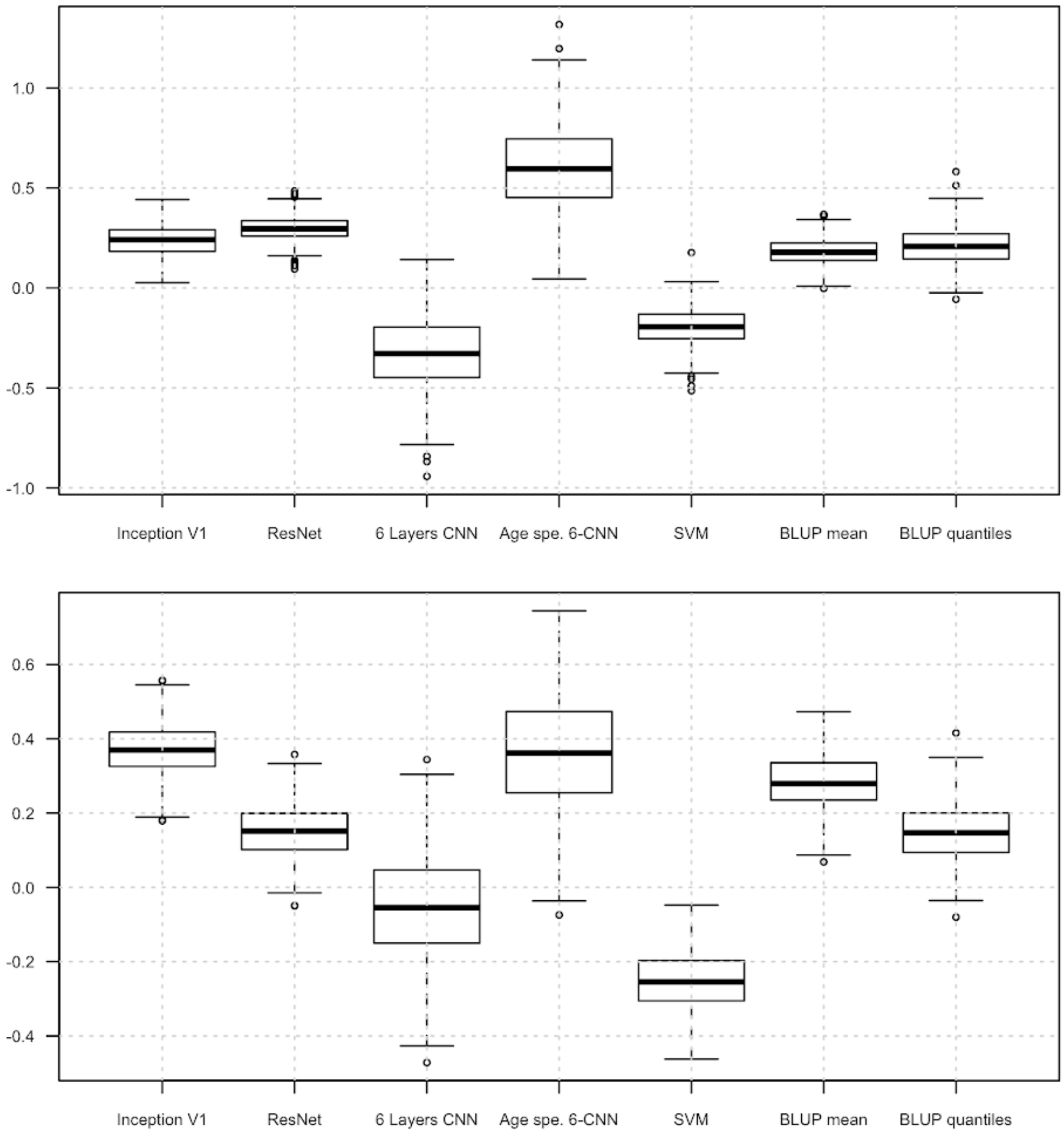

Supplementary Figure 1: distribution of linear weights in ensemble learning for PAC challenge 1.

The different panels correspond to the five folds used in the analysis. In each fold, we present the distribution of weights, estimated by linear regression on ~265 participants, over 500 bootstrap iterations (see methods).

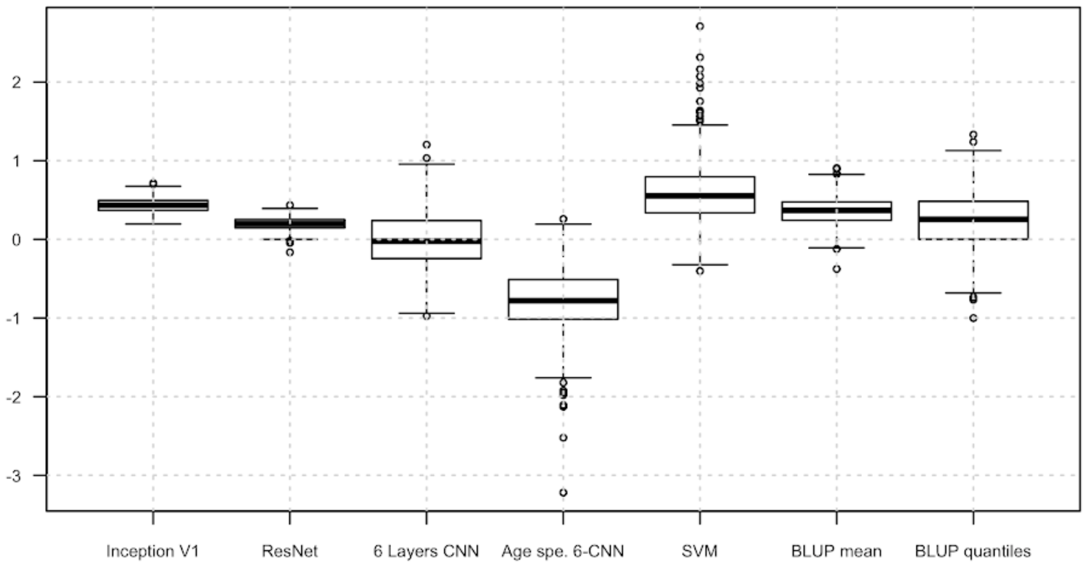
2
1
0
-1
-2
-3
Inception V1
ResNet
6 Layers CNN
Age spe. 6-CNN
SVM
BLUP mean
BLUP quantiles

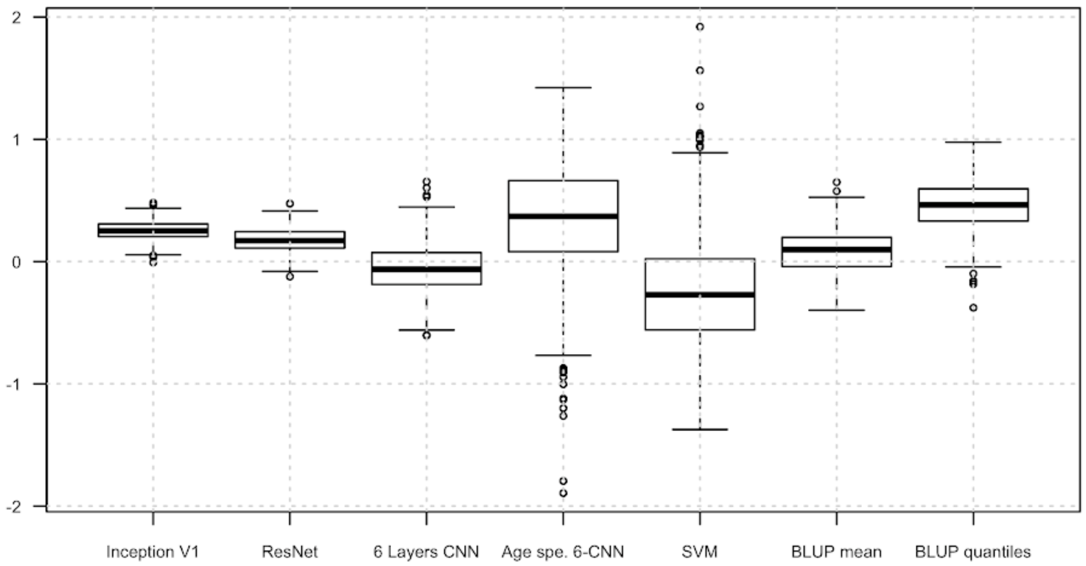
2
1
0
-1
-2
Inception V1
ResNet
6 Layers CNN
Age spe. 6-CNN
SVM
BLUP mean
BLUP quantiles

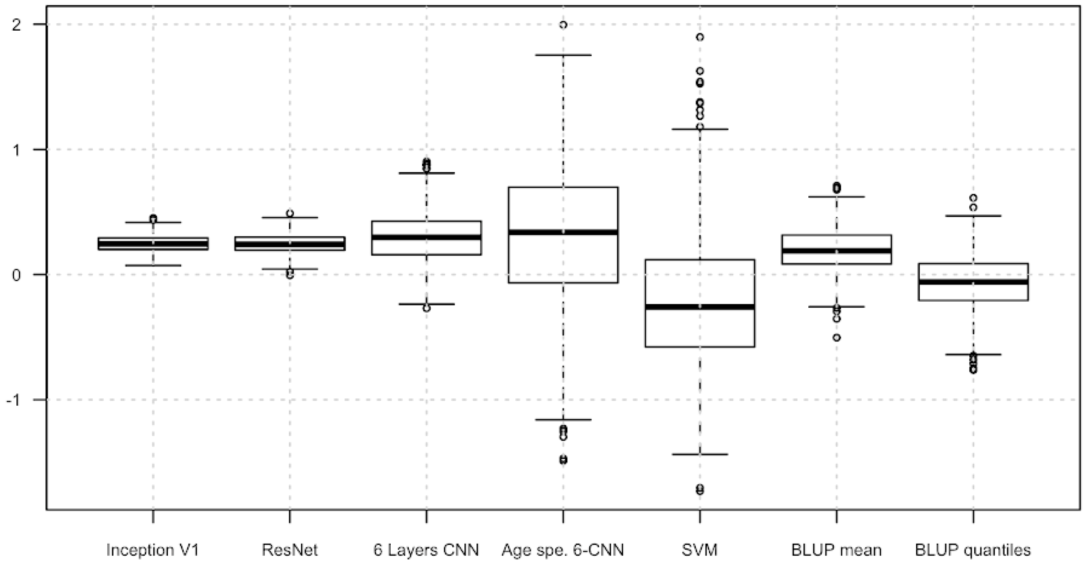
2
1
0
-1
Inception V1
ResNet
6 Layers CNN
Age spe. 6-CNN
SVM
BLUP mean
BLUP quantiles

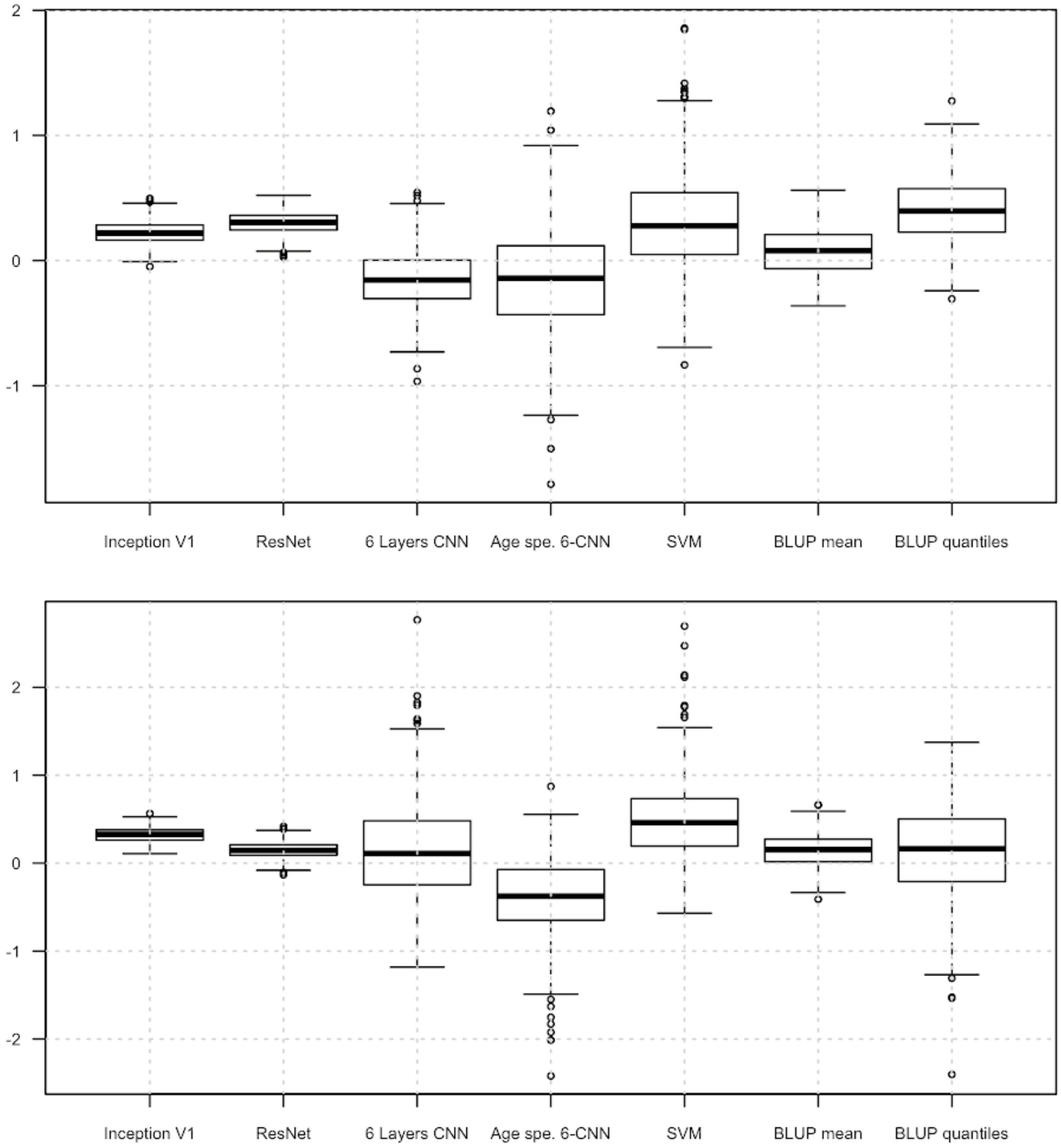

Supplementary Figure 2: distribution of linear weights in ensemble learning for PAC challenge 2.
The different panels correspond to the five folds used in the analysis. In each fold, we present the distribution of weights, estimated by linear regression on ~265 participants, over 500 bootstrap iterations (see methods).
